# Statin-Induced Immune-Mediated Necrotizing Myopathy: An Increasingly Recognized Inflammatory Myopathy

**DOI:** 10.7759/cureus.7963

**Published:** 2020-05-05

**Authors:** Oana P Stroie, Joshua Boster, Luke Surry

**Affiliations:** 1 Internal Medicine, Uniformed Services University of the Health Sciences, Bethesda, USA; 2 Internal Medicine, Brooke Army Medical Center, Fort Sam Houston, San Antonio, USA; 3 Internal Medicine, San Antonio Uniformed Services Health Education Consortium, San Antonio, USA

**Keywords:** necrotizing myopathy, myositis, immune-mediated, anti-hmgcr

## Abstract

Statin-induced immune-mediated necrotizing myopathy, also known as anti-3-hydroxy-3-methylglutaryl-coenzyme A reductase (anti-HMGCR) myopathy, is an inflammatory myopathy that is triggered by statin exposure and persists after statin discontinuation. It is a rare side effect of statins, distinct from the more commonly recognized statin-induced myalgia, that is challenging to diagnose and treat. We describe a case of anti-HMGCR myopathy in a 59-year-old male with a prior history of statin intolerance presenting with markedly elevated creatinine kinase, myoglobinuria, and one month of progressive proximal muscle weakness after restarting atorvastatin 10 months prior to admission. High-dose glucocorticoids led to rapid clinical improvement, although the patient relapsed upon tapering. Remission was attained at three months after combination therapy with azathioprine, intravenous immunoglobulin, and a prolonged prednisone taper.

## Introduction

Statin drugs, ubiquitous in middle and advanced age for the management of atherosclerosis, are increasingly associated with autoimmune disease. Statins are generally considered to be safe and effective with a benign side effect profile, although myalgia is a common complaint. Statin-induced myalgias are due, in part, to the inhibition of cholesterol biosynthetic pathways that may, in turn, lead to upregulation of cholesterol biosynthesis-related genes that subsequently results in reduced skeletal muscle size, protein synthesis, and muscle atrophy [1]. This phenomenon is generally self-limited. Alternatively, inflammatory myopathies are caused by autoimmune-mediated damage to muscle tissue leading to proximal muscle weakness. Among the most well-known inflammatory myopathies are polymyositis, dermatomyositis, and inclusion body myositis. The presence of extramuscular involvement, autoantibodies, and histological examination are used to differentiate between the different subtypes [2]. However, there is a rising incidence of a novel subset of statin-induced immune-mediated necrotizing myopathy (IMNM) that is distinct from statin-induced myalgias and previously described inflammatory myopathies. IMNM is rare with an estimated incidence of approximately two to three of every 100,000 patients treated with statins [3]. 

IMNM is an increasingly recognized form of inflammatory myopathy. There are two subtypes that are distinguished based on specific autoantibodies: anti-3-hydroxy-3-methylglutaryl-coenzyme A reductase (anti-HMGCR) and anti-signal recognition particle (SRP). Patients with anti-HMGCR myopathy generally have a history of statin use and are typically older than patients with anti-SRP myopathy [4].

In anti-HMGCR myopathy, the immune system becomes sensitized to increased levels of HMG-CoA reductase that is upregulated by statins, which results in a necrotizing immune-mediated injury to cells that express this enzyme [5]. These autoantibodies continue to persist long after statin discontinuation and result in ongoing tissue damage. Similar to other inflammatory myopathies, statin-induced anti-HMGCR myopathy is distinguished by a specific antibody assay. To our knowledge, there are no controlled trials that have compared the different therapeutic regimens utilized for anti-HMGCR myopathy, but the best evidence is for immunosuppression. This case illustrates the diagnostic pitfalls and response to treatment for this rare condition.

## Case presentation

A 59-year-old Caucasian man with a history of hypertension, hyperlipidemia, and type 2 diabetes mellitus presented to emergency department after preprocedural labs, for a scheduled liver biopsy to further evaluate persistently elevated liver-associated enzymes (LAEs), revealed a significantly elevated creatine kinase (CK) concerning for rhabdomyolysis. He complained of extreme fatigue and muscle soreness for the past month with difficulty climbing stairs and raising his arms. He denied any preceding fever, chills, rashes, joint pain, dysphagia, diplopia, or any recent systemic infections. He had no history of autoimmune disease, malignancy, or hereditary disease. Ten months prior to presentation, the patient restarted atorvastatin 40 mg, following a one-year discontinuation secondary to elevated LAEs.

Physical exam findings were notable for reduced muscle strength symmetrically with hip flexion 2/5, knee flexion 4/5, shoulder abduction 4/5, and elbow flexion 4/5. Gait was stable and he was able to walk unassisted. There was 1+ pitting edema in the bilateral lower extremities. No fasciculation, atrophy, or swelling of the affected muscles was noted. No skin rashes. Heart, lung, and abdominal examinations revealed no abnormalities and vital signs were within normal ranges.

Laboratory evaluation was notable for significantly elevated CK 17,145 IU/L (N: 24-170 IU/L), elevated aspartate transaminase 449 U/L (N: 5-40 U/L), alanine transaminase 397 U/L (N: 4-41 U/L), and normal alkaline phosphatase. Urinalysis revealed 78 red blood cells and myoglobinuria 993 ng/mL (N: 0-13 ng/mL). Renal function panel, thyroid panel, and complete blood count were within normal limits. Urine drug screen, antinuclear antibody, and myositis panels were negative.

**Figure 1:**
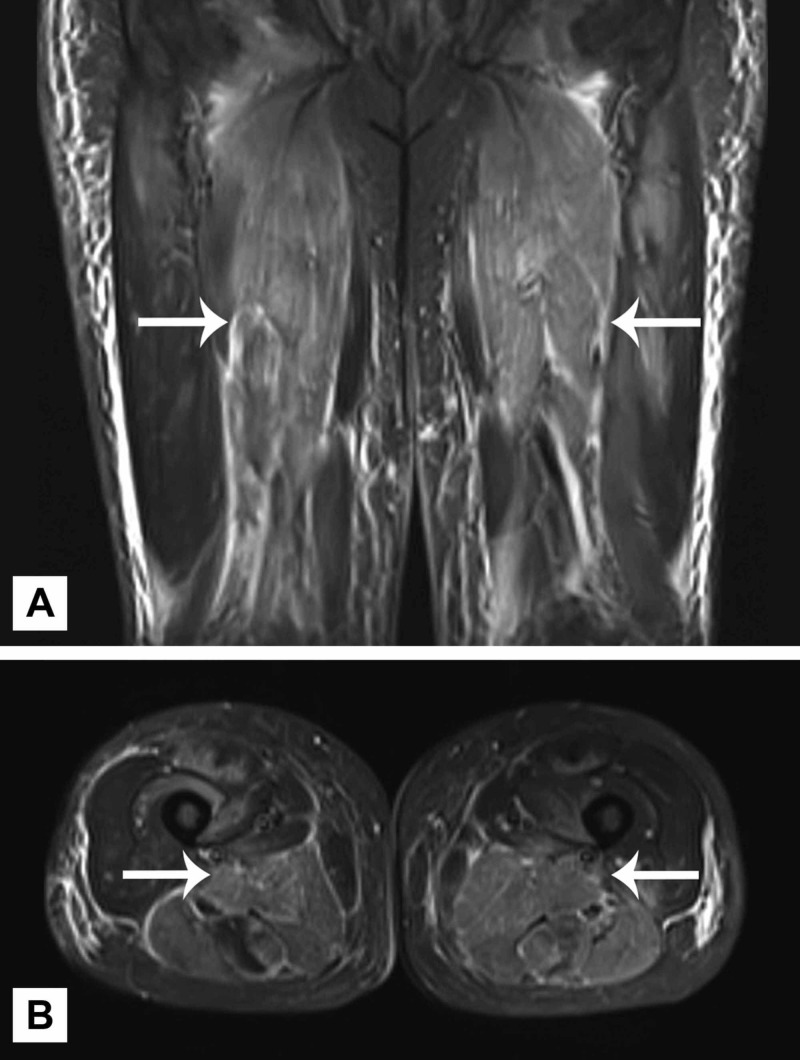
Coronal (A) and axial (B) STIR MRI sequences showing diffuse myositis. STIR, short-TI inversion recovery.

The patient was admitted to the general medical floor with the initial diagnosis of statin induced rhabdomyolysis based on an elevated CK and myoglobinuria. Atorvastatin was discontinued, and aggressive intravenous (IV) hydration was initiated. The patient initially improved with a precipitous drop in CK after the first 24 hours. Beyond the first day, CK elevation persisted despite ongoing aggressive IV hydration. On hospital day 6, IMNM was considered, and the anti-HMGCR antibody test and MRI of thighs were obtained for further evaluation. MRI of the thighs revealed diffuse myositis throughout hamstrings, quadriceps, abductor, and gluteal muscles with trace surrounding soft tissue edema (Figure 1). Anti-HMGCR assay results eventually revealed strongly positive anti-HMGCR antibodies at >200 (N: 0-20) after hospital discharge. 

Based on the MRI evidence of diffuse myositis, failure to improve despite aggressive hydration, and an otherwise negative serological evaluation, the patient was started empirically on 80 mg of prednisone for suspected statin-induced IMNM. He demonstrated a rapid response to glucocorticoid therapy and was discharged home with plan to continue prednisone 80 mg by mouth daily with slow taper over the following month.

**Figure 2:**
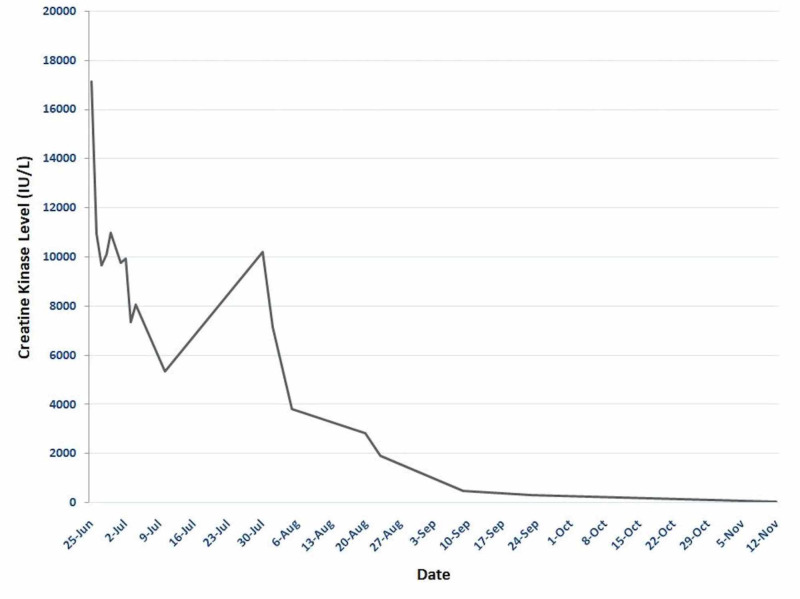
Creatine kinase levels and response to therapy over time. Jun, June; Jul, July; Aug, August; Sep, September; Oct, October; Nov, November.

Prednisone therapy led to a significant improvement in symptoms; however, initial tapering to 40 mg resulted in worsening symptoms and a readmission to the medical floor. Combination therapy was initiated with prednisone 80 mg daily, azathioprine, and intravenous immunoglobulin (IVIG). IVIG infusions were administered every four weeks and azathioprine was titrated up to 200 mg daily coinciding with a slow prednisone taper. The patient responded well, with gradual symptomatic resolution and improvement in muscle strength with normalization of his CK occurring over several months (Figure 2). 

## Discussion

Inflammatory myopathies are a family of rare autoimmune diseases that often pose a diagnostic challenge due to their relative unfamiliarity and broad differential of symptom presentation. The ubiquitous use of statins for the treatment of cardiovascular disease has unmasked a new subset of inflammatory myopathies, anti-HMGCR myopathy that is associated with statin exposure. Failed response to initial treatment in patients presenting with proximal muscle weakness and history of statin use should prompt consideration of alternative diagnoses to include inflammatory myopathies. Unlike other inflammatory myopathies, anti-HMGCR myopathy has not been found to be significantly associated with malignancy. It can manifest at any time in people with current or past history of statin use, and in some cases, in the absence of statin use secondary to exposure to statin-like compounds found in foods and dietary supplements like oyster mushrooms and red yeast rice [6].

Inflammatory myopathies are characterized by progressive proximal muscle weakness and markedly elevated CK. The inflammatory etiology in dermatomyositis, polymyositis, and inclusion body myositis is due to a T-cell-mediated immune response against muscle fibers that can be identified on muscle biopsy. In contrast, IMNMs are due to an antibody-mediated immune reaction against muscle fibers that can be identified on biopsy by the presence of macrophages and necrotic fibers undergoing phagocytosis [1,4]. Muscle biopsy is not necessary for the diagnosis as it is less specific for IMNMs than immunoassays [4].

The mainstay of treatment for statin-induced IMNM is immunosuppression. Anti-HMGCR myopathy is difficult to treat due to continued antibody production long after discontinuation of the offending agent [4]. There are no established guidelines for the management of anti-HMGCR myopathy in regard to both optimal treatment regimen and duration. Our patient was started on a high dose of prednisone with a prompt response to therapy followed by a relapse upon initial tapering. Increasingly, evidence suggests combination therapy with high-dose glucocorticoids, methotrexate, azathioprine, or IVIG to be more efficacious in sustaining remission [7]. In patients with diabetes and other cardiovascular comorbidities, a corticosteroid free induction and maintenance may be preferred to minimize the risk of steroid complications [8]. In some cases, even monotherapy with IVIG may be considered [9].

Our patient sustained complete remission within the first three months after initiation of combination therapy with prednisone, IVIG, and azathioprine. This case demonstrates the importance of following serum CK levels closely to monitor response to therapy prior to glucocorticoid tapering. 

## Conclusions

Anti-HMGCR myopathy is an IMNM that is associated with statin use. This diagnosis remains relatively rare, and it presents with several diagnostic and therapeutic challenges. Optimal management is unclear, but aggressive immunosuppression is effective, although early glucocorticoid tapering in our patient was associated with a recurrence of symptoms. Further research is needed to identify optimal treatment regimens and duration of therapy for these patients.
